# High dose escalation of intracoronary adenosine in the assessment of fractional flow reserve: A retrospective cohort study

**DOI:** 10.1371/journal.pone.0240699

**Published:** 2020-10-15

**Authors:** Chien-Boon Jong, Tsui-Shan Lu, Patrick Yan-Tyng Liu, Mu-Yang Hsieh, Shih-Wei Meng, Ching-Chang Huang, Hsien-Li Kao, Chih-Cheng Wu

**Affiliations:** 1 Department of Internal Medicine, National Taiwan University Hospital, Hsin-Chu Branch, Hsin-Chu, Taiwan; 2 Department of Mathematics, National Taiwan Normal University, Taipei, Taiwan; 3 Division of Cardiology, Department of Internal Medicine and Cardiovascular Center, National Taiwan University Hospital, Taipei, Taiwan; 4 College of Medicine, National Taiwan University, Taipei, Taiwan; 5 Cardiovascular Center, National Taiwan University Hospital, Hsin-Chu Branch, Hsin-Chu, Taiwan; 6 Institute of Biomedical Engineering, National Tsing-Hua University, Hsin-Chu, Taiwan; 7 Institute of Cellular and System Medicine, National Health Research Institutes, Zhunan, Taiwan; The Open University, UNITED KINGDOM

## Abstract

Maximal hyperaemia for fractional flow reserve (FFR) may not be achieved with the current recommended doses of intracoronary adenosine. Higher doses (up to 720 μg) have been reported to optimize hyperaemic stimuli in small dose-response studies. Real-world data from a large cohort of patients is needed to evaluate FFR results and the safety of high-dose escalation. This is a retrospective study aimed to evaluate the safety and frequency of FFR ≤0.8 after high-dose escalation of intracoronary adenosine. Data were extracted from the medical databases of two university hospitals. Increasing doses (100, 200, 400, 600, and 800 μg) of adenosine were administered as intracoronary boluses until FFR ≤0.8 was achieved or heart block developed. The percentage of FFR ≤0.8 after higher-dose escalation was compared with those at conventional doses, and the predictors for FFR ≤0.8 after higher doses were analysed. In the 1163 vessels of 878 patients, 402 vessels (34.6%) achieved FFR ≤0.8 at conventional doses and 623 vessels (53.6%) received high-dose escalation. An additional 84 vessels (13.5%) achieved FFR ≤0.8 after high-dose escalation. No major complications developed during high-dose escalation. Borderline FFR (0.81–0.85) at the conventional dose, stenosis >60%, and triple-vessel disease increased the likelihood of FFR ≤0.8 after high-dose escalation, but chronic kidney disease decreased it. For vessels of borderline FFR at conventional doses, 46% achieved FFR ≤0.8 after high-dose escalation. In conclusion, High-dose escalation of intracoronary adenosine increases the frequency of FFR ≤0.8 without major complications. It could be especially feasible for borderline FFR values near the 0.8 diagnostic threshold.

## Introduction

Coronary angiography is not a perfect tool for functional assessment of coronary artery disease. It may under- or overestimate the functional severity, especially in cases of intermediate stenosis. The fractional flow reserve (FFR) measurement facilitates better understanding of the clinical and physiological significance of angiographic coronary stenoses. FFR is now considered the standard for evaluation of the ischemic potential and expected benefit from revascularization procedures [1], and is recommended in patients with 40–90% stenosis [2]. Nonetheless, the measurement of FFR has to be standardised to avoid technical- or operator-related variations. During FFR procedures, achievement of maximal hyperaemia is a key requisite for an accurate calculation of FFR. Without maximal hyperaemia, FFR is overestimated and stenosis severity is underestimated, leaving ischemic stenosis untreated [3]. Although intravenous infusion of adenosine is considered the gold standard, intracoronary injection of adenosine is used more commonly in daily practice [4]. Despite the wide adoption of intracoronary adenosine, the optimal dose remains up for debate. Currently, doses of 100 μg in the right coronary artery (RCA) and 200 μg in the left coronary artery (LCA) are recommended, based on dose-response data from normal coronary arteries [5]. However, higher doses (up to 720 μg) have been reported to optimize hyperaemic stimuli in small dose-response studies [6, 7]. All the dose-response studies enrolled relatively small numbers of patients with insufficient power to detect differences. Real-world data from a sufficient number of patients is needed to evaluate the FFR results and safety after high-dose escalation of intracoronary adenosine. Our institution employs a protocol of increasing doses (100, 200, 400, 600, and 800 μg) of adenosine intracoronary boluses for hyperaemic stimuli unless the ischemic threshold (FFR ≤0.8) was achieved, or complete atrioventricular block or sinus pause developed. The aim of this study was to report the safety and frequency of FFR ≤0.8 after high-dose escalation of intracoronary adenosine in our institution.

## Materials and methods

**Fig 1:**
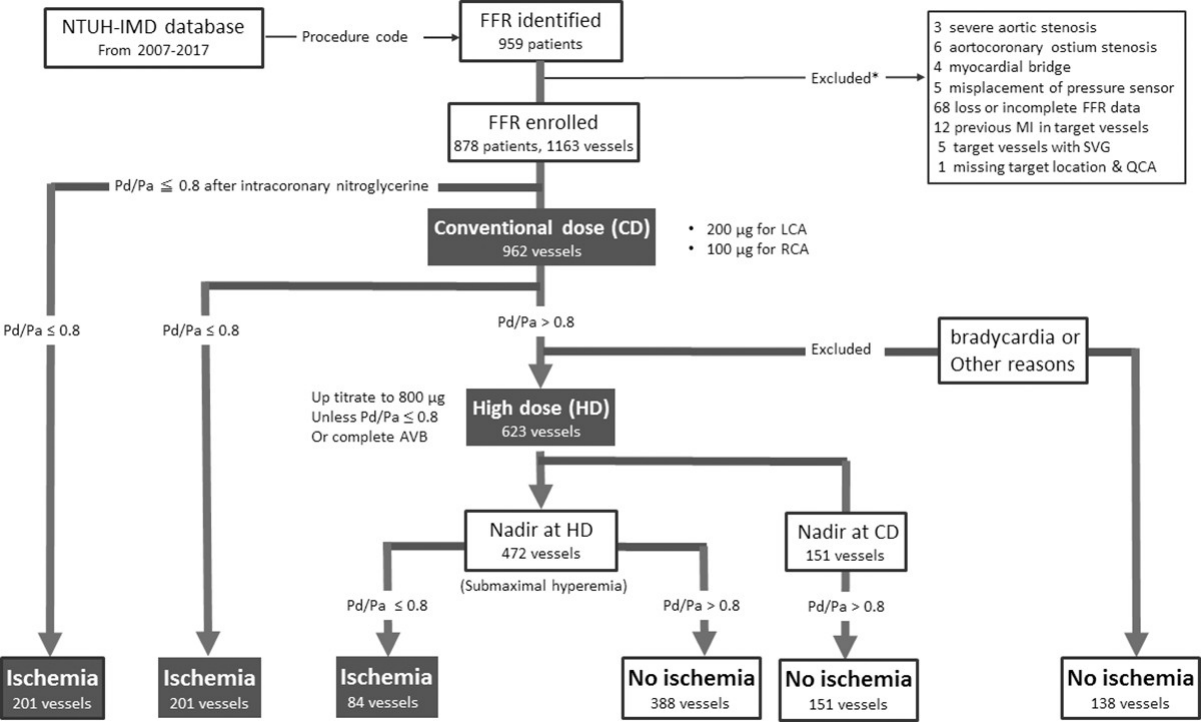
Diagram of patient flow. In the 1163 vessels, one-hundred thirty-eight vessels with negative FFR at conventional dose did not proceed to high dose intracoronary adenosine. * Severe aortic stenosis counts as per patient, otherwise count as per vessel. Abbreviations: FFR: fractional flow reserve; LCA: left coronary artery; NTUH-IMD: National Taiwan University Hospital integrated medical database; RCA: right coronary artery; SVG: saphenous vein graft.

This is a retrospective cross-sectional study conducted in National Taiwan University Hospital, a 2,600-bed tertiary referral centre, and its affiliated hospital Hsinchu Branch, a 900-bed hospital, both in northern Taiwan. The study subjects were selected according to medical claims for pressure-monitoring guide wire (Certus or Aeris pressure wire; St. Jude Medical Inc., St. Paul, MN, USA). Between March 2007 and September 2017, 959 patients were initially identified from the database. After a medical record review, 81 patients were excluded due to the following criteria: severe aortic stenosis, aortocoronary ostium stenosis, myocardial bridge, saphenous vein graft, previous myocardial infarction at target vessels, misplacement of pressure sensor, missing target location, or incomplete FFR data. The final cohort enrolled 878 patients with 1163 vessels (Fig 1).

FFR was measured as previously described [8]. Briefly, after a pressure monitoring guidewire was advanced distal to the coronary artery stenosis, 100 or 200 μg of nitroglycerine was administered intracoronary. Hyperaemia was obtained through the intracoronary administration of adenosine. FFR was defined as the minimum ratio of the simultaneously recorded mean arterial pressure distal (Pd) to the stenosis and mean aortic pressure at the tip of the guiding catheter (Pa); we avoided measuring during ectopic heartbeats or the beat after atrioventricular block. In our institution, the dosage of adenosine was increased by increments to 100, 200, 400, 600, and 800 μg. This escalation was terminated when FFR decreased to ≤0.8 or when complete atrioventricular block or sinus pause developed. An FFR ≤0.8 was used as the diagnostic threshold of “positive” for ischemia. In this study, intracoronary administration of 100 μg in the RCA or 200 μg in the LCA was defined as the “conventional-dose” adenosine. When intracoronary doses above the conventional-dose adenosine were escalated, the regimen was defined as “high-dose” adenosine.

The data of the study population were extracted from the integrated medical database of National Taiwan University Hospital (NTUH-iMD). The NTUH-iMD contains all information, such as demographics, diagnosis, medication, procedures, laboratory results, clinical patient notes, nursing notes, and death records, of inpatient and outpatient visits to the NTUH since 2006. Structured data were extracted from the database according to the identification number and date of FFR measurement. Unstructured data, such as comorbidity, clinical presentation, and both FFR and QCA detailed data, were extracted from the medical record, which was recorded by an experienced cardiologist. To optimize the accuracy of information from NTUH-iMD and data accuracy, chart reviews were conducted as needed. This study was approved by the institutional review board (IRB No.201709074RIND) of National Taiwan University Hospital, and the need for informed consent was waived.

Descriptive statistics for the baseline characteristics were generated either on a per-patient or per-vessel basis, depending on the clinical measurements. The McNemar test was used to compare the dependent proportions of ischemic vessels during hyperaemia with conventional- and high-dose intracoronary adenosine. Multi-collinearities among potential explanatory variables were investigated to avoid model-burdening correlations between variables. To account for correlations among vessel measurements from the same subject, the generalized estimating equations method [9] was used to evaluate the associations between positive FFR in higher doses of intracoronary adenosine and potential predictors, assuming an exchangeable correlation structure. We first examined each variable at the significance level of 0.05 and then explored several full models for the positive FFR in higher doses, conditional on age, sex, body mass index and other significant factors from the univariate analyses. A stepwise variable-selection algorithm was used for more complete exploration of the data and the variables adjusted in the final model included age, sex, BMI, CKD, TVD, lesion stenosis>60%, target vessel with diffuse or tandem lesion, and borderline FFR (0.81–0.85) at standard dose. All data analyses were performed using SAS version 9.4 (SAS Institute, Cary, NC, USA).

## Results

The characteristics of the patients and vessels are summarized in Tables 1 & 2. The mean age of the study subjects was 65 years, and 78% were men. They were referred for coronary angiography for the following reasons: chronic stable angina (88%), acute coronary syndrome (12%), or heart failure (<1%). Regarding the target vessels, 57% were in the left anterior descending (LAD) artery, 19% in the left circumflex artery, 21% in the RCA, and 1% in the left main coronary.

**Table 1:** Characteristic of the patients.

**Demographic data**	
Age, year	65.2 (11.2)
Sex (female)	196 (22)
Body mass index, kg/m^2^	26.1 (3.7)
Current smoker	165 (19)
**Comorbidities**	
Hypertension	660 (75)
Diabetes mellitus	335 (38)
Hyperlipidaemia	469 (53)
Chronic kidney disease*	247 (28)
Atrial fibrillation/flutter	66 (7.5)
Heart failure	109 (12)
Prior ST-segment elevation myocardial infarction	29 (3.3)
Prior peripheral arterial disease	41 (4.7)
Prior stroke	51 (5.8)
**Presentations**	
Acute coronary syndrome	137 (12)
ST elevation myocardial infarction^†^	7 (1)
Non-ST elevation myocardial infarction	79 (7)
Unstable angina	51 (4)
Stable angina	1021 (88)
Heart failure	5 (0.4)
**Medications**	
Aspirin	680 (77)
P2Y12 inhibitor	410 (47)
Statin	573 (65)
Beta blocker	546 (62)
**Extent of coronary artery disease**^‡^	
1 vessel	279 (32)
2 vessels	279 (32)
3 vessels	320 (36)

Values are given as number (%), mean (SD), or median (IQR).

* Chronic kidney disease defined as estimated GFR<60 ml/min per 1.73 m^2^ or end-stage renal disease.

^†^One patient received FFR assessment after 8 days of acute myocardial infarction. Another patient received FFR assessment at non-culprit vessel after 1 day of acute myocardial infarction. The other two patients (including 5 target vessels) received FFR assessment after 2 months of acute myocardial infarction.

^‡^Extent of disease defined as index diagnosis with ≥50% luminal stenosis in main trunk or major branches of epicardial vessels.

**Table 2:** Characteristic of the target vessels.

**Target vessel**	
Left main	7 (1)
Left anterior descending	669 (57)
Left circumflex/obtuse marginal branch	226 (19)
Right coronary/Posterior descending/Posterolateral	247 (21)
Ramus intermedius	26 (2)
Diagonal branch	8 (1)
**Target Location**	
Ostium (Left anterior/circumflex only)	56 (5)
Proximal	392 (34)
Middle	417 (36)
Distal	223 (19)
Branches	75 (6)
**Lesion distribution**	
Focal	858 (74)
Diffuse	94 (8)
Tandem	221 (19)
**Target lesion stenosis**	
30–49%	42 (4)
50–60%	845 (73)
60–70%	236 (20)
71–90%	40 (3)
**FFR distribution**	
FFR ≤ 0.8	486 (42)
FFR 0.81–0.9	473 (41)
FFR > 0.9	204 (17)
Mean FFR value	0.82 (0.09)
Median FFR value	0.83 (0.77, 0.89)

Values are given as number (%), mean (SD), or median (IQR). Abbreviations: FFR, functional flow reserve.

**Fig 2:**
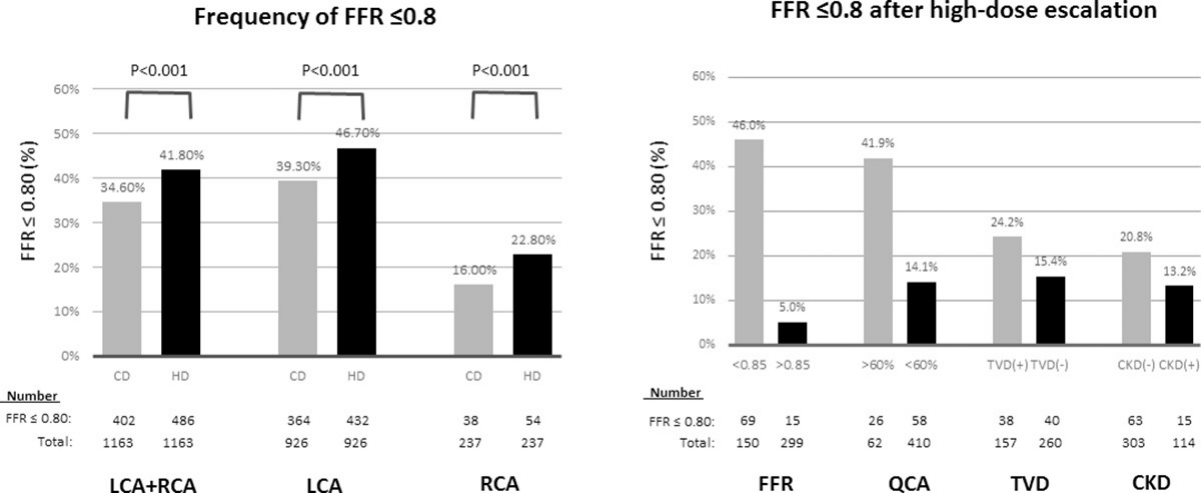
Effect of high-dose intracoronary adenosine stratified by functional, angiographic, and clinical factors. **Left**: Comparison of the proportion of FFR≤0.80 during hyperaemia with conventional dose and high dose intracoronary adenosine in all vessels (total), left coronary artery and right coronary artery. **Right**: Percentage of vessels re-allocated into FFR≤0.80 after high-dose intracoronary adenosine administration, stratified by fractional flow reserve value, stenosis severity, triple-vessel disease and chronic kidney disease. Abbreviations: CD: conventional dose; CKD: chronic kidney disease; FFR: fractional flow reserve; HD: high dose; LCA: left coronary artery; QCA: Quantitative coronary angiography; RCA: right coronary artery; TVD: triple-vessel disease.

**Fig 3:**
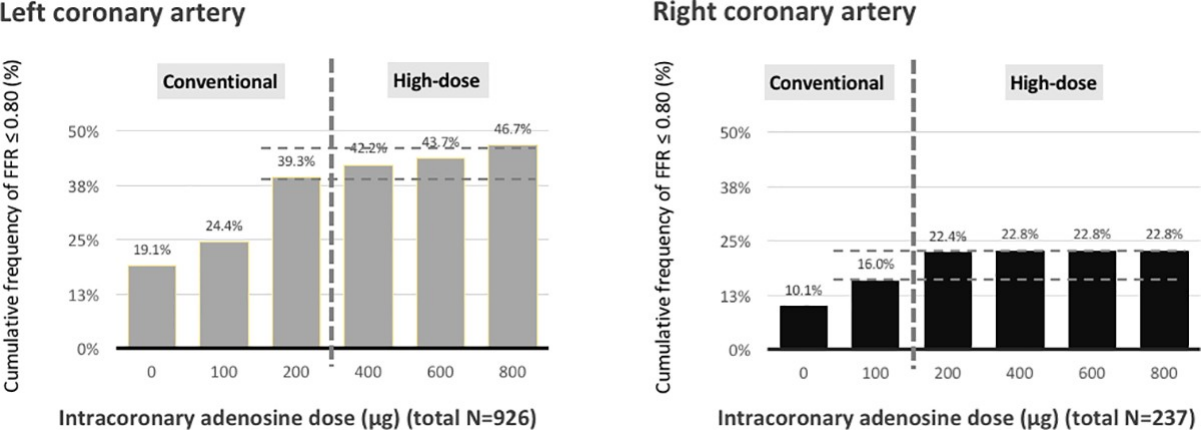
Frequency of vessels with FFR ≤ 0.80 by adenosine dosage. The cumulative rate of FFR≤0.80 increased gradually (after the conventional dose) in the left coronary artery while high-dose escalating, whereas the cumulative rate plateaued after a 200 μg adenosine injection in the right coronary artery. Bar: percentage of vessels recorded with FFR ≤ 0.8 at this dose; solid line: cumulative percentage of vessels recorded FFR ≤ 0.8 at this dose. Abbreviations: FFR: fractional flow reserve.

**Fig 4:**
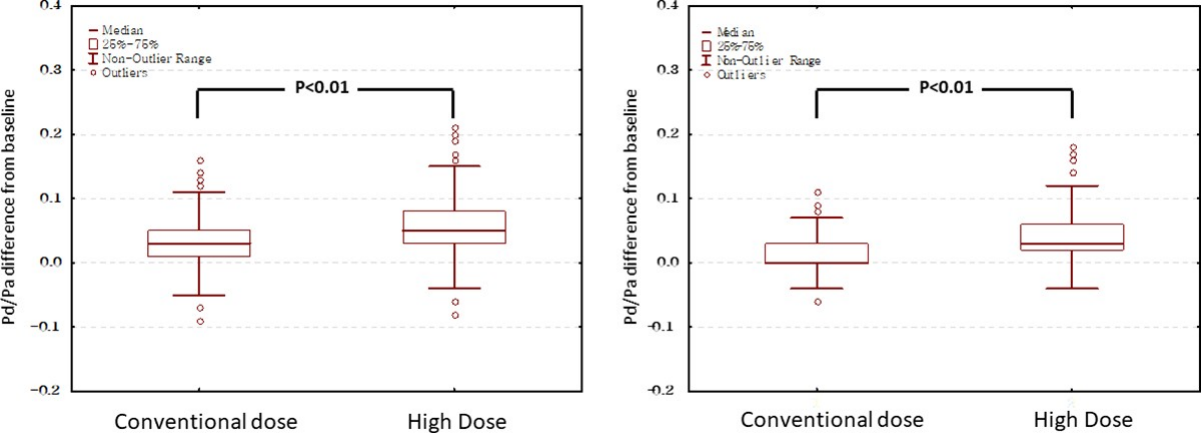
The difference of Pd/Pa from baseline. The difference of Pd/Pa from baseline was increased after high-dose escalation of intracoronary adenosine than that by using the conventional doses of adenosine.

FFR was measured in 1163 vessels of 878 patients. After intracoronary boluses of nitroglycerine, Pd/Pa≤0.80 was achieved in 201 vessels. During the initial hyperaemic stimuli with conventional dose of adenosine, another 201 vessels achieved an FFR≤0.8. Escalation of adenosine beyond the conventional dose was not continued in 138 vessels due to bradycardia or other reasons. Escalation of adenosine was continued in the remaining 623 vessels. The nadir of Pd/Pa decreased in 472 vessels (submaximal hyperaemia in conventional dose) but remained consistent in the other 151 vessels. In the 472 vessels with submaximal hyperaemia, escalation of adenosine up to 800 μg maximally revealed an additional 84 vessels achieving an FFR≤0.8 (Fig 1). After high-dose escalation, the overall frequency of FFR≤0.8 increased from 34.6% to 41.8% (Fig 2, Left). The percentage of vessels achieving an FFR≤0.8 at various doses of adenosine is displayed in Fig 3. It increased from 39.3% to 46.7% for LCA and from 16.0% to 22.8% for RCA. The difference of Pd/Pa from baseline was increased after high-dose escalation of intracoronary adenosine as compared to that after using conventional dose, both in left coronary artery (median of difference, 0.05 vs. 0.03, p<0.01) and right coronary artery (median of difference, 0.02 vs. 0 p<0.01) (Fig 4).

Four patients had complications related to the FFR procedures (0.3%), but only one was related to adenosine administration: 1 patient received temporary cardiopulmonary massage due to a transient sinus pause after intracoronary administration of 100 μg adenosine in RCA and 3 patients had complications related to wire manipulation, including a broken tip of the pressure wire, dissection, and distal embolization.

In univariate analysis, borderline FFR at the conventional dose (0.81–0.85), stenosis >60%, diffuse or tandem lesions, TVD, and stenosis of the LAD were associated with a positive response at high-dose escalation. In multivariate analysis, borderline FFR at the conventional dose, stenosis >60%, TVD, and non-CKD patients remained associated with newly provoked positive response at high-dose escalation (Table 3). Among these factors, borderline FFR values at the conventional dose best predicted FFR≤0.8 at high-dose escalation. In this study, one third of the patients had FFR ranging between 0.81 to 0.85 at conventional doses. For patients with borderline FFR values, 46% achieved FFR≤0.8 after high-dose escalation. In contrast, only 5% of patients with FFR>0.85 at conventional doses achieved FFR≤0.8 after high dose escalation. The frequency of FFR≤0.8 after high-dose escalation is displayed in Fig 2, right, stratified by relevant predicting factors.

**Table 3:** Predictors of FFR≤0.8 at high-dose escalation.

	Crude		Adjusted ^a^	
Factors	OR (95% CI)	P value	OR (95% CI)	P value
Age	0.98 (0.96–1.00)	0.058	-	-
Male sex	0.97 (0.54–1.76)	0.921	-	-
BMI	1.02 (0.92–1.09)	0.540	-	-
Diabetes	1.19 (0.74–1.94)	0.476	-	-
CKD*	0.54 (0.29–0.99)	0.045	0.37 (0.18–0.74)	0.005
Heart failure	1.10 (0.54–2.21)	0.798	-	-
TVD	1.68 (1.04–2.71)	<0.001	2.43 (1.31–4.51)	0.005
LAD stenosis	2.04 (1.25–36.35)	0.005	-	-
Stenosis > 60%	4.43 (2.52–7.79)	<0.001	3.39 (1.54–7.47)	0.003
Diffuse/tandem	3.27 (1.92–5.57)	<0.001	-	-
FFR (0.81–0.85)	16.1(8.72–29.8)	<0.001	18.3 (9.66–34.6)	<0.0001

Abbreviations: BMI, body mass index; CKD: chronic kidney disease; FFR, functional flow reserve; LAD, left anterior descending artery; TVD: triple vessel disease.

^a^ Risk factors considered in the full model using stepwise algorithm included age, sex, BMI, CKD, TVD, lesion stenosis>60%, target vessel with diffuse or tandem lesion, and borderline FFR (0.81–0.85) at standard dose. Factor of target vessel with LAD stenosis was avoided due to collinearity found between LAD stenosis and borderline FFR at standard dose.

* CKD defined as estimated GFR<60 ml/min per 1.73 m^2^ or end-stage renal disease.

## Discussion

This study reports on the safety and FFR results after high-dose escalation of intracoronary adenosine in a large cohort of patients in routine clinical practice. We found that, among 623 vessels with FFR >0.80 at conventional intracoronary adenosine doses, applying a dose escalation strategy reclassified 13.5% of these arteries as having FFR values ≤0.80 and did not precipitate any major complications. Some angiographic (diameter stenosis >60%, TVD), physiological (FFR 0.81–0.85), and clinical (CKD) factors were associated with FFR≤0.8 after high-dose escalation.

Maximal hyperaemia is paramount for the determination of FFR. Randomized controlled trials used intravenous infusions of adenosine at a rate of 140 μg/kg/min [1]. Intracoronary administration of adenosine is a possible alternative, but the optimal dose is still a matter of debate. Recently, higher doses of intracoronary adenosine (100 μg for the RCA and 200 μg for the LCA) were recommended, according to dose-response data from 30 angiographically normal coronary arteries [5, 8]. The coronary flow velocity measurement showed that more than 95% of maximal hyperaemia could be achieved at this dose, but higher doses were not suggested [5]. However, these dosage recommendations were challenged by the results of higher doses of intracoronary adenosine (up to 600–720 μg) reported in some studies [6, 7, 10]. In 50 intermediate lesions, Leone et al. [6] reported that only intracoronary doses up to 600 μg yielded FFR values similar to intravenous adenosine. In 108 lesions, Lopez et al. [7] found that 600 μg of adenosine rendered more physiologic ischemia classifications than 60 μg and 20% more than intravenous infusion (37.6% vs. 31.5%). De Luca et al. [10] used similar doses of intracoronary boluses in 50 lesions, showing greater detection of ischemia at higher doses (51.2% with 720 μg vs. 38% with 120 μg). Our study shown that the cumulative rate of FFR≤0.80 increased gradually in the left coronary artery while high-dose escalating, whereas the cumulative rate plateaued after a 200 μg adenosine injection in the right coronary artery.

Our data provided clinical validation of the findings reported from the studies of high-dose adenosine. We demonstrated that high-dose escalation identified FFR values ≤0.80 in 13.5% of vessels with initially negative FFR results (>0.80). The frequency of re-allocation is generally in line with the fact that 8%–10% of cases failed to achieve maximal hyperaemia at conventional doses of adenosine [11, 12]. The conflict with current recommended doses might be due to the following reasons. First, the sample sizes in previous dose-response studies were relatively small and not powered to detect a 10%–15% difference in positive FFR. Second, high-dose escalation might help overcome the variability of adenosine response in certain patients [13, 14]. Third, in cases of severe microvascular disease, FFR may be higher than expected and underestimate epicardial stenosis severity [15–17]. The current recommended doses were based on studies of near-normal coronary arteries [5]. There may be a higher prevalence of coronary microvascular dysfunction in arteries with flow-limiting coronary stenoses.

Safety is another concern with intracoronary administration of high-dose adenosine. Because of the retrospective nature of our study, minor adverse effects, such as chest pain, dyspnoea, flushing, or nausea were not routinely recorded in the FFR reports or catheterization reports. Asymptomatic atrioventricular block might be under-reported as well. Nonetheless, our cohort demonstrated that no patient experienced major complications (therapy required, permanent sequelae, or death) secondary to the high-dose escalation. Only one patient received temporary cardiopulmonary massage due to a transient sinus pause after injecting conventional (100μg) rather than high dose adenosine into the RCA. Of note, the use of high intracoronary doses of adenosine is not uncommon in daily practice, especially in the setting of no reflow during coronary interventions [18]. The step-by-step escalation of adenosine doses avoided a further increase of doses in cases of AV block and prevented excessive prolonged bradycardia which may have caused complications or needed therapy. Our data suggested that high-dose escalation of IC adenosine was generally safe. However, approximately one-fifth of patients with FFR >0.80 were excluded from receiving higher doses of adenosine due to concerns of bradycardia and/or other reasons following administration of standard IC adenosine doses. This is a relevant consideration when attempting to draw conclusions about the safety of high-dose adenosine in this study.

According to our analysis, the most powerful determinants of FFR≤0.8 at high-dose escalation was borderline FFR values (0.81–0.85) at conventional doses. By analysing reproducible data from the DEFER study [19], Petraco et al. [20] found that the diagnostic agreement between repeated FFR measurements fell within the range of borderline values (0.75–0.85), reaching nadir around the 0.80 cut-off point. In vessels with FFR values ranging from 0.77 to 0.83, a repeated measurement may reclassify the FFR value to the opposite side of the 0.80 revascularization threshold in 20% of cases. Our data suggested that biological variability to hyperaemic response may be a more relevant cause of uncertainty, rather than the measurement variability. A previous study demonstrated that coronary blood flow fluctuated widely at submaximal hyperaemic status, which could be resolved by escalating the dosage of adenosine [13]. Consequently, escalating the hyperaemic stimuli for borderline FFR may be a rational approach to overcome biological variability.

Regarding angiographic parameters, the severity of stenosis, distribution of lesions, target vessel involved, and number of diseased vessels were all associated with positive FFR results during high-dose escalation, in line with previous studies [21, 22]. Similar to the results of previous publications, our study found that atherosclerotic risk factors, prior myocardial infarction, or heart failure did not affect the FFR results at high-dose escalation [12, 13]. More target vessels with a LAD lesions received higher-dose escalation than the target vessels with an RCA stenosis. Higher-dose escalation for a LAD lesion also had a higher chance of re-allocation than that for an RCA lesion (HR = 2.04, 95% CI = 1.25–36.4). This finding is relevant because deferral of LAD revascularization based on FFR assessment might be associated with a poor cardiovascular outcome [23]. Interestingly, patients with chronic kidney disease (CKD) had a lower positive rate at high-dose adenosine, as described previously in a multicentre prospective registry [24]. In patients with advanced kidney diseases, severe left ventricular hypertrophy, heavy coronary calcification, and diabetic microangiopathy are more common and thus may attenuate the hyperaemic response to adenosine [25, 26]. Further studies are needed to clarify the response of FFR measurements in patients with CKD.

Based on our results, the least precise measurement of FFR is right around the cut-point of 0.80 at conventional dose. We estimate that nearly one-third of all initial FFR are located at the border zone area of 0.81–0.85 and 46% of these vessels will achieve FFR≤0.8 after high-dose escalation. For the two third of vessels with FFR >0.85, high-dose escalation might not be needed because only 5% of them will change to FFR≤0.8. In such circumstances, physicians may or may not justify high-dose escalation based on more information, such as clinical symptoms, risk-benefit profile, and financial burden on the patients. In patients with acute myocardial infarction, the FFR response of a non-culprit vessel might be varied by the timing of examination and its relation to the acute event [27]. Studies also demonstrated that ACS patients had higher subsequent ischemic events even in the borderline FFR range [28]. Our study had only limited number and information of the ACS patients. The efficacy and safety of high-dose escalation in ACS patients should be justified after more evidence available.

Some limitations of this study should be addressed. First, this was a retrospective study; hence, some data were not available for review or from medical records. Second, the study is an analysis of routine clinical practice but not a dose-response study. Therefore, variations in the increments of dosage existed, and it was not possible to show a continuous dose-response effect. Nonetheless, unselected patients were enrolled, and the results are representative of clinical scenarios. Third, owing to the retrospective nature of the study, there is lack of “positive control” with intravenous adenosine infusion to identify some technical issues. Fourth, operators were not blinded to the different doses of adenosine. However, given the fully automated measurement, this should not significantly bias the results. Fifth, the outcome benefit of revascularization in these newly allocated positive response group needs further investigation. Finally, the clinical relevance of FFR≤0.8 under high coronary blood flow might be different from that under conventional doses. Since other independent evidence of ischemia or validation with intravenous adenosine were not available, the prognostic significance of FFR decline under high-dose adenosine needs to be clarified in future prospective outcome studies.

## Conclusions

The study suggested that high-dose escalation beyond the current recommended doses of intracoronary adenosine increases the frequency of FFR≤0.80 without serious complications. High-dose escalation is especially warranted for a borderline FFR result at conventional dose.
